# Computer-based cognitive training for older adults: Determinants of adherence

**DOI:** 10.1371/journal.pone.0219541

**Published:** 2019-07-10

**Authors:** Merita Turunen, Laura Hokkanen, Lars Bäckman, Anna Stigsdotter-Neely, Tuomo Hänninen, Teemu Paajanen, Hilkka Soininen, Miia Kivipelto, Tiia Ngandu

**Affiliations:** 1 Public Health Promotion Unit, National Institute for Health and Welfare (THL), Helsinki, Finland; 2 Department of Psychology and Logopedics, Faculty of Medicine, University of Helsinki, University of Helsinki, Helsinki, Finland; 3 Aging Research Center, Karolinska Institutet, Stockholm University, Stockholm, Sweden; 4 Department of Psychology, Umeå University, Umeå, Sweden and Department of Social and Psychological Sciences, Karlstad University, Karlstad, Sweden; 5 Neurocenter/Neurology, Kuopio University Hospital, Kuopio, Finland; 6 Research and Service Centre for Occupational Health, Finnish Institute of Occupational Health, Helsinki, Finland; 7 Institute of Clinical Medicine/Neurology, University of Eastern Finland, Kuopio, Finland; 8 Division of Clinical Geriatrics, Center for Alzheimer Research, NVS, Karolinska Institutet, Stockholm, Sweden; Nathan S Kline Institute, UNITED STATES

## Abstract

The possibilities of computer-based cognitive training (CCT) in postponing the onset of dementia are currently unclear, but promising. Our aim is to investigate older adults´ adherence to a long-term CCT program, and which participant characteristics are associated with adherence to the CCT. This study was part of the Finnish Geriatric Intervention Study to Prevent Cognitive Impairment and Disability (FINGER). Participants were 60-77-year-old individuals with increased dementia risk, recruited from previous population-based studies. The participants included in this study (n = 631) had been randomized to receive a multi-domain lifestyle intervention, including CCT. The measure of adherence was the number of completed CCT sessions (max = 144) as continuous measure. Due to a substantial proportion of participants with 0 sessions, the zero inflated negative binomial regression analyses were used to enable assessment of both predictors of starting the training and predictors of completing a higher number of training sessions. Several cognitive, demographic, lifestyle, and health-related variables were examined as potential predictors of adherence to CCT. Altogether, 63% of the participants participated in the CCT at least once, 20% completed at least half of the training, and 12% completed all sessions. Previous experience with computers, being married or cohabiting, better memory performance, and positive expectations toward the study predicted greater odds for starting CCT. Previous computer use was the only factor associated with a greater number of training sessions completed. Our study shows that there is a large variation in adherence to a long-lasting CCT among older adults with an increased risk of dementia. The results indicate that encouraging computer use, and taking into account the level of cognitive functioning, may help boost adherence to CCT.

## Introduction

The number of people living with dementia and Alzheimer’s disease (AD) continues to grow [1]. Developing interventions to delay the onset of cognitive decline/dementia is of increasing interest [2]. Computer-based cognitive training (CCT) is a potentially important tool for individuals at risk of dementia [3]. Compared to traditional cognitive training, CCT allows the level of challenge to be more easily individualized and adapted as a result of training progression. Further, CCT is easily scalable, and training data can be automatically recorded. [4]. Cognitive training in healthy older adults and those at risk of dementia has been linked to benefits in cognitive functioning, mostly in small and short-term trials, even if the evidence is mixed, especially regarding transfer of the cognitive benefits [3–9].

CCTs are labor intensive, and participants must adequately adhere to training to attain a sufficient training dosage. Currently, there is no golden standard for defining good or poor adherence; and in practice, there is probably a dose-response relation between adherence and intervention outcome. Adherence has mostly been studied in the context of medication use, and it has been estimated that adherence to long-term therapy for chronic illnesses averages around 50% [10]. Less is known about adherence to non-pharmacological interventions, including CCTs. Different demographic, psychosocial, and cognitive factors may affect the ability of the individual to participate in and benefit from a given intervention, and understanding these factors is crucial for wider implementation of interventions in the population at large. However, to our knowledge, no study has investigated determinants of adherence to CCTs.

In a non-computerized intervention, to improve memory and memory self-efficacy among well-educated middle-aged and older adults, adherence was predicted by better health, higher education, and higher self-efficacy before training [11]. In another study among older adults with mild cognitive impairment (MCI), women and participants with better cognition had higher overall adherence to interventions focusing on either physical exercise, cognitive ability, combined physical and cognitive exercise, or social activity [12], but age and education did not affect adherence. The combined cognitive-physical training group showed the lowest adherence rate [12]. Cognitive deficits including impairments in episodic memory and executive dysfunctions may make it difficult to initiate and commit to long-term, labor-intensive interventions, including cognitive training [13,14]. Depressive symptoms, social withdrawal and low self-efficacy may also reduce adherence [13,15].

This study investigated adherence to a long-lasting CCT among older adults with an increased dementia risk from the general population, and identified participant characteristics associated with adherence to training.

## Methods

### Study design

The current study was part of the Finnish Geriatric Intervention Study to Prevent Cognitive Impairment and Disability (FINGER). The protocol of FINGER has been described earlier [16]. In brief, FINGER is a multi-centered randomized controlled trial aiming to lower the risk of cognitive impairment in the elderly at an increased risk of cognitive decline, conducted in Finland (ClinTrials identifier NCT01041989). The 2-year, multi-domain intervention consisted of a combination of nutritional guidance, exercise, cognitive training and social activity, and management of metabolic and vascular risk factors. Participants in the intensive intervention group received all four components of the intervention. The components of the multi-domain intervention were initiated in a step-wise manner to facilitate adherence to each component. The cognitive training component was the last to start, approximately 4–6 months after randomization. Persons in the control group received regular health advice. The primary outcome was cognitive performance measured with the modified Neuropsychological Test Battery (NTB) [17]. The screening phase ran from September 2009 until December 2011. The intensive intervention period was completed in 2014, and the first results indicate that the multi-domain intervention had a beneficial effect on cognition [18]. FINGER has been approved by the Coordinating Ethics Committee of the Helsinki and Uusimaa Hospital District, Finland.

### Participants

FINGER participants were recruited from persons who had participated in population-based, non-communicable disease risk-factor surveys [19,20]. To be invited, the person had to be aged 60–77 years at the beginning of the study and have Cardiovascular Risk Factors, Aging and Incidence of Dementia (CAIDE) risk score of 6 points or higher, indicating presence of some risk factors for dementia [21]. At the screening visit, participants´ cognitive functioning was assessed with the Consortium to Establish a Registry for Alzheimer´s Disease (CERAD) neuropsychological test battery [22]. To be included, participants had to fulfill at least one of the following criteria: (1) Word List Learning task (10 words x 3) ≤ 19 words; or (2) Word List Savings ≤ 75%; or (3) Mini Mental State Examination (MMSE) ≤ 26/30 points. These criteria selected persons with cognitive performance at the mean level or slightly lower than expected for age according to Finnish population norms [23]. Exclusion criteria were conditions affecting engagement in the intervention: present malignant diseases, major depression, dementia/substantial cognitive decline according to clinical interview, MMSE < 20, symptomatic cardiovascular disease, re-vascularization within one year, severe loss of vision, hearing or communicative ability, conditions preventing cooperation, as judged by the local study physician, as well as coincident participation in any other intervention trial.

Participants were randomized in either the intensive multi-domain intervention or the control group (1:1). Those randomized into the intervention group (n = 631) form the basis of the current study.

### Computer-based cognitive training and adherence

The computer-based cognitive training program was developed from protocols previously shown to be effective in RCTs [24–27]. The training targeted four cognitive functions known to be critical to cognition in general (mental speed, working memory, executive functions, and episodic memory).

The cognitive intervention started with an introduction phase consisting of six group sessions during which the participants were trained to use the CCT and there were also educational discussions on memory-related themes. Participants were then asked to continue the CCT independently at their homes. As not all participants had computers at home, they were also given the possibility to come and train at study centers. In addition, there were four visits for testing the progress of CCT. The training comprised eight tasks: a one-back task to train mental speed; maintenance of visuo-spatial locations task for working memory; updating tasks (spatial, verbal, numerical), a set-shifting task to train executive processes; a relational (word triplets to be bound together during encoding), and a classic memory-game task to train episodic memory. The independent training consisted of two blocks of 72 sessions each, delivered three times/week for 10–15 minutes/session (until two presented tasks were completed). The two blocks lasted six months each, with an interval of 3–6 months in between. The CCT program introduced different tasks in a sequential order so that the participant had to complete the presented tasks (two tasks at each session) to be able to move forward. Computer-based exercises enabled an individually adjusted increase in difficulty levels. Activity and performance in the program were registered automatically to our database. Adherence to the training was measured as the number of completed training sessions out of a maximum of 144 sessions.

### Cognitive assessment

Cognitive performance at the baseline was measured by the modified Neuropsychological Test Battery (NTB), known to be a reliable and sensitive measure for mild cognitive changes in Alzheimer´s disease (AD) [17] and complemented with tests measuring executive function to also capture changes typical for vascular cognitive impairment [28]. The memory domain included Visual Paired Associates immediate and delayed; Logical Memory, immediate and delayed from the Wechsler Memory Scale–Revised [29]; and Word List Learning and delayed recall of the CERAD test battery [22]. The executive function domain included Category Fluency [22], Digit Span [29], Concept Shifting (Condition C) [30], Trail Making (shifting score B-A) [31], and a shortened 40-stimulus version of the original Stroop Test (interference score 3–2) [32]. The processing speed domain included Letter Digit Substitution [33], Concept Shifting (condition A) and Stroop (condition 2). NTB was administered by trained psychologists who were not conducting the cognitive intervention. Zero-skewness log transformation was applied to skewed NTB components. Scores for executive functioning, processing speed, and memory were obtained by averaging individual NTB component z-scores. A minimum number of necessary NTB components was set to 3/5 for executive functioning, 2/3 for processing speed, and 3/6 for memory.

### Measures of other variables

Height, weight, and blood pressure were measured by trained study nurses at the baseline visit. Fasting total serum cholesterol and plasma glucose concentrations were analyzed. Apolipoprotein E (ApoE) genotype was determined [34] and dichotomized into ApoE ɛ4 carriers (1 or 2 ɛ4alleles) and non-carriers. The Zung Depression Scale [35] was used to evaluate depressive symptoms (total score ranges from 20–80, with higher scores indicating more depressive symptoms). The level of physical functioning was assessed with the Short Physical Performance Battery (SPPB) [36] and dichotomized (1 = less than 12 points indicating some difficulties, 2 = 12 points, indicating no difficulties). Data on subjective health, subjective memory and subjective mood were collected using 5-point Likert scales, which were dichotomized (1 = average or below; 2 = above average). Number of self-reported chronic diseases (possible range 0–18) and number of visits to a general practitioner during the previous year were analyzed as continuous variables. Data on frequency of drinking alcohol to become drunk was dichotomized as being drunk at least monthly, or less than monthly. Smoking habits were divided to current smokers (regular or occasional) and non-smokers (both never and former smokers). Frequency of leisure-time physical activity lasting at least 20 minute and causing sweating and breathlessness was grouped into low (once a week or less often), moderate (2–3 times per week) or high (4 times a week or more often). Leisure activity was assessed by asking how often participants attended 11 different activities (reading books or newspapers, doing crossword puzzles, writing, playing board games or cards, playing musical instrument or singing, participating in organized groups, studying, doing handicrafts, gardening, babysitting, and doing voluntary work). Participants reported the frequency of participation as “daily”, “4–6 times per week”, “2–3 times per week”, “once weekly”, “2–3 times per month”, “a couple of times per year or less”, or “never”. We summed the participation in all activities into a single variable and categorized the number of activities per week into 3 categories with equal frequencies (less than 12 activities per week, 12–17 activities per week, 18 or more activities per week). Individuals´ expectations for participation in the study was assessed using a 5-point Likert scale, which was dichotomized (1 = cannot say or negative, 2 = positive or very positive). Data on previous computer use was collected with the question “Do you use a computer?” (yes/no). Adherence to other intervention domains was based on the number of individual and group visits with the study nutritionist for the dietary component, number of gym visits for physical activity, and number of intervention visits to the study nurse and physician for vascular risk-factor management.

### Statistical analyses

Statistical analyses were performed using Stata Data Analysis and Statistical Software (StataCorp 2009. Stata Statistical Software: Release 11.2. College Station, TX: StataCorp LP). Differences in predictor variables between trained and non-trained participants were analyzed using chi square and t tests, and associations between predictor variables and number of training sessions among those who trained at least once were analyzed with t tests or Spearman correlations. The outcome variable was number of training sessions. As a substantial proportion (37%) of participants did not log onto the training program even once, and to take into account the possibility that mean and variance of the distribution of the training sessions are not equal, the zero-inflated version of the negative binomial regression (ZINB) was used to analyze predictors of adherence. ZINB estimates two coefficients for each variable. One coefficient estimated the association between predictors and no training versus any training. The other coefficient estimates the association between each variable and the number of training sessions among those that trained. Results for these coefficients are reported as incidence rate ratios (IRR), with 95% confidence intervals (CI). Only variables that were significant at p < .10 in the bivariate analyses were included in the full multivariate model. In addition to the full model, a reduced model was estimated. Here, models with all combinations of different parameters included in the full model were estimated. The model with the lowest Bayesian information criterion was considered to be the best fitting model. Participants with any missing data were excluded from the corresponding analyses (n = 69).

Additional analyses were conducted 1) including also adherence to other intervention domains among the explanatory variables, to take into account overall adherence to multi-domain intervention and 2) excluding previous computer use from the model due to a large effect of computer use in the main analyses.

## Results

### Participant characteristics

**Fig 1:**
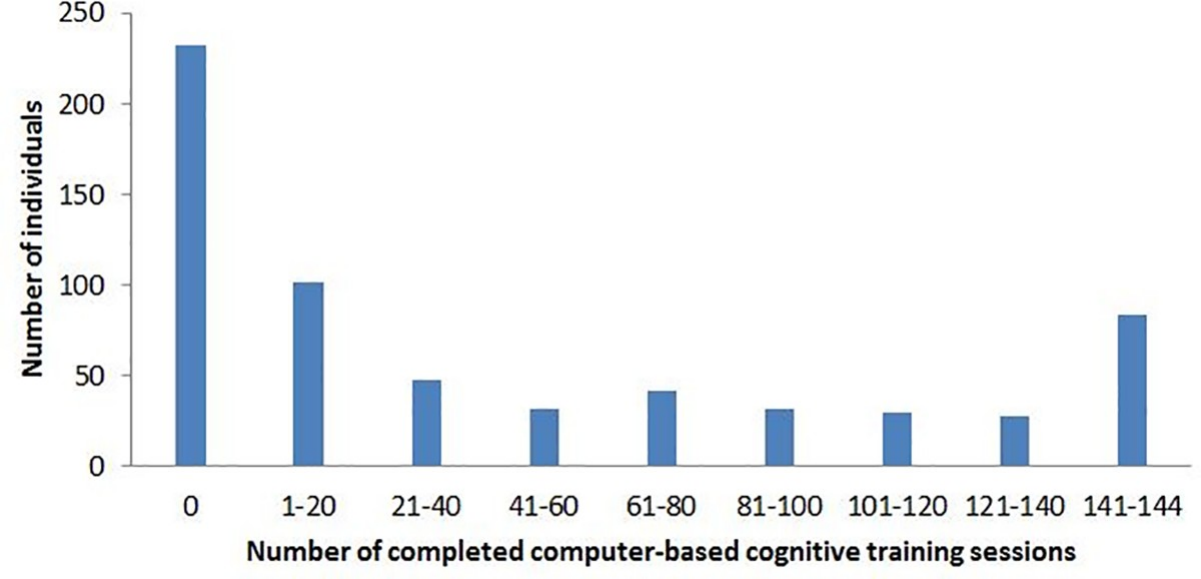
Distribution of the participants according to the number of completed training sessions.

The mean age of participants (n = 631) was 69.5 years and 54.7% of them were men. Of these, 63% participated in the CCT at least once, 20% completed at least half of the cognitive training, and 12% completed all this training. The mean number of CCT sessions was 45.7 (SD 54.95), and median 15 (95% CI 6–23). Distribution of training sessions is shown in Fig 1.

Table 1 shows baseline characteristics for the trained and non-trained groups. Participants who underwent at least one training session were included in the trained group. Trained participants were younger, more likely to be women and more likely to be married or cohabiting, and they had higher education than the non-trained group. Also, among the trained participants, there were more computer users at the baseline, cognitive performance at the baseline was better, they were in better physical condition, and they tended to have more positive expectations toward participating in the study compared to the non-trained participants. Non-trained participants had more depressive symptoms compared to trained participants, and their subjective health was slightly poorer.

**Table 1:** Baseline characteristics for trained and non-trained participants.

	All	Trained participants n = 398	Non-trained participants n = 233	*p*-value
**Demographic characteristics**				
Baseline age, years	631	68.8 (4.4)	70.7 (4.8)	**<0.001**
Number of women	631	229/398 (57.5)	116/233 (49.8)	**0.06**
Education, years	621	10.3 (3.4)	9.5 (3.6)	**0.01**
Married/Cohabiting	626	315/395 (79.7)	144/231 (62.3)	**<0.001**
Previous computer use	622	269/397 (67.8)	67/225 (29.8)	**<0.001**
**Medical/lifestyle factors**				
Serum total cholesterol, mmol/L	630	5.2 (1.0)	5.2 (1.0)	0.96
Fasting plasma glucose, mmol/L	630	6.1 (0.9)	6.1 (0.7)	0.94
Systolic blood pressure, mm Hg	628	139.9 (15.9)	140.08 (17.1)	0.51
Body-mass index, kg/m^2^	627	28.4 (0.2)	28.1 (0.3)	0.34
Number of current smokers	627	33/397 (8.3)	28/230 (12.2)	0.12
Being drunk at least monthly	613	49/392 (12.5)	27/218 (12.4)	0.92
Depressive symptoms (Zung)	606	34.8 (7.8)	37.03 (8.5)	**<0.001**
ApoE Ɛ4 carriers	590	122/374 (32.6)	67/216 (31.3)	0.69
Number of chronic diseases	629	2.4 (1.6)	2.5 (1.5)	0.87
Good level of physical functioning (SPPB)	605	163/396 (41 .1)	61/209 (29.2)	**0.004**
Physical activity	625			0.66
low		117 (29.6)	72 (31.3)	
moderate		142 (36.0)	87 (37.8)	
high		136 (34.4)	71 (30.9)	
Leisure time activity	629			0.12
low		125 (31.5)	90 (38.8)	
moderate		125 (31.5)	72 (31.0)	
high		147 (37.0)	70 (30.2)	
**Self-reported health status**				
Good health	627	247/396 (62.4)	122/231 (52.8)	**0.02**
Good memory	629	184/397 (46.3)	95/232 (40.9)	0.19
Good mood	629	301/397 (75.8)	166/232 (71.6)	0.24
Physician visits last 12 months	613	2.2 (2.2)	2.5 (2.6)	0.14
**Cognition**				
NTB total score	631	0.1 (0.5)	-0.2 (0.6)	**<0.001**
Executive functioning	631	0.0 (0.6)	-0.2 (0.7)	**<0.001**
Processing speed	631	0.1 (0.8)	-0.2 (0.8)	**<0.001**
Memory	631	0.0 (0.7)	-0.2 (0.7)	**<0.001**
**Attitude to participating the study**				
Positive expectations	629	382/398 (96.0)	204/231 (88.3)	**<0.001**

Data is n, n/N (%), or mean (SD). Participants who underwent at least one training session were included in the trained group. SPPB = Short Physical Performance Battery. NTB = neuropsychological test battery. Scores on the NTB total score, and on executive functioning, processing speed, and memory are mean values of Z scores of the cognitive tests included for each cognitive outcome, with higher scores indicating better performance.

In bivariate analyses among those who trained at least once (n = 398), younger age, female gender, previous computer use, being a non-smoker, and having lower fasting glucose were associated with greater number of completed CCT sessions (results not shown). Persons with missing data for any of the predictor variables (n = 69) also completed less of the CCT (mean number of completed sessions 15.9, SD 4.7) compared to those with complete data (mean 49.4, SD 2.3), p<0.001. Otherwise they were similar to participants with complete data (n = 562), except that those with missing data had fewer positive expectations toward participating in the study, they performed slightly worse in the processing speed domain, and were more often women.

### Multivariate results

The results of the multivariate analyses are shown in Table 2. All variables significant at p< 0.10, or better, in bivariate analyses were included in the full ZINB model. In the full model, previous experience with computers, being married or cohabiting, better memory performance, and positive expectations toward the study predicted greater likelihood for starting the training. Previous computer use was also positively associated and smoking was negatively associated with the number of completed training sessions. In the reduced ZINB model, results remained unchanged, except that smoking was not included in the best-fitting model.

**Table 2:** Predictors of adherence to CCT.

	Full model (BIC = 4762.538)						Reduced model (BIC = 4641.225)					
	Starting the CCT (zero-inflation part)			Number of CCT sessions (negative-binomial part)			Starting the CCT (zero-inflation part)			Number of CCT sessions (negative-binomial part)		
Variables	IRR	CI	*p*	IRR	CI	*p*	IRR	CI	*p*	IRR	CI	*p*
Education	.951	.88–1.03	.201	.992	.95–1.03	.657						
Age	.985	.94–1.04	.576	.976	.95–1.00	.059						
Sex (women)	.967	.59–1.59	.895	1.219	.95–1.56	.116						
Marital status (not co-habiting)	.511	.31-.85	**.010**	1.051	.80–1.39	.724	.479	.30-.77	**.002**			
Memory/NTB	1.887	1.28–2.78	**.001**	.934	.78–1.12	.468	1.716	1.22–2.42	**.002**			
Executive function/NTB	.821	.52–1.30	.403	.968	.77–1.21	.780						
Processing speed/NTB	1.106	.76–1.60	.594	1.004	.84–1.20	.960						
Previous computer use	6.581	3.87–11.18	**<0.001**	1.439	1.11–1.86	.**005**	6.239	3.88–10.03	**<0.001**	1.418	1.13–1.78	**.001**
Subjective health	.975	.61–1.56	.917	1.01	.79–1.28	.961						
Study expectations	4.624	1.88–11.3	.**001**	1.265	.69–2.34	.452	4.442	1.85–10.69	**.001**			
Physical condition (SPPB)	1.541	.94–2.54	.089	.963	.77–1.21	.748						
Depressive symptoms (Zung)	.991	.96–1.02	.537	.995	.98–1.01	.543						
Fasting glucose	1.058	.81–1.38	.679	.919	.82–1.03	.163						
Current smoking	.887	.43–1.81	.742	.636	.44-.93	**.018**						

Multivariate zero-inflated negative binomial regression model was used (n = 562) to estimate the incidence-rate ratios (IRR) and the corresponding confidence intervals (CI). The higher IRR in the “starting the CCT” column indicates that the variable is linked to a greater likelihood of completing at least one session of CCT. Higher IRR in the column “number of CCT sessions” indicates that the variable is linked to completing a greater number of training sessions.

In additional analyses, when participation in other intervention components (diet, physical activity, and vascular risk factor management) was also included, the associations that were observed in the main analyses remained unchanged. In addition, participation in diet and vascular interventions were associated with a greater likelihood to start CCT, and participation in physical activity and vascular interventions with a greater amount of completed CCTs. A second set of additional analyses excluding the computer use from the full model did not change the effect of other variables.

## Discussion

An increasing interest exists in developing CCT to maintain cognitive function and delay the onset of dementia. Our study suggests that the degree of adherence to a CCT may vary across participants: 20% of participants completed at least half and 12% completed all of the CCT sessions, whereas 37% did not train at all. To our knowledge, the present CCT is the longest and most intensive conducted so far, and it has been suggested that the level of adherence may be higher in short-term and single-domain CCTs [12]. Previous use of computers, better memory, being married/cohabiting, and positive study expectations were independently associated with the greater probability of starting the CCT. Previous computer use was the main determinant of the number of CCTs completed after the training was initiated. As computer use is rapidly increasing among older adults, the adherence level to similar interventions may be expected to increase in the years to come.

Our findings are in line with a previous non-computerized cognitive training trial, suggesting that better cognitive functioning may affect, but chronological age may not affect adherence [12]. Earlier studies have reported conflicting results concerning education [11,12], and in our study, education did not predict adherence. Contrary to previous studies [11,12], we did not observe an association between sex or health status and adherence. Positive expectations toward the study were strongly associated with starting the training. Persons who were married or cohabiting were more likely to engage in CCT. It may be an indicator that social support is important for adherence, but this has not been explored previously. Our results further indicate that starting the training is the critical step. Thereafter, the number of training sessions completed was unrelated to all participant characteristics, except computer use.

Previous computer use was identified as an important determinant of both starting the training and completing a greater amount of training sessions. The exact reasons for this could not be investigated in our study, but possible reasons include lack of necessary computer skills and lack of an owned or at least easily accessible computer.

Our results show that CCT is feasible even in this older age group, but there were substantial between-person differences in degree of adherence. Nearly two-thirds of the participants trained at least a little. A smaller proportion completed the entire training program. The FINGER CCT was much more extensive and longer lasting than most previous CCT trials [4]. According to the pre-specified definition, 50% participation was considered adherent [16]. This amount was achieved by 20% of the participants. Currently, it is unclear how much training is needed to achieve cognitive benefits. The positive results of the overall FINGER multi-domain intervention suggest that even lower levels of adherence might be sufficient. However, the contributions of each of the intervention domains on the overall outcome is difficult to disentangle.

The CCT was designed to be cognitively challenging to be effective. This may have reduced adherence among some of the participants. Previously, it has been suggested that more demanding interventions (including more than one domain) may result in an increased drop-out rate [12]. Thus, it is important to note the multi-domain and long-lasting nature of FINGER when judging the adherence rates. CCT was the last intervention component in our study, and some participants may have felt reluctant to add additional activities to the ongoing physical activity and nutritional interventions. We have previously reported that self-reported participation in other intervention domains of FINGER was higher compared to CCT [18]. In the current analyses, participation in other intervention domains was associated with greater CCT adherence.

It is still unknown how much CCT or other intervention activities are needed for optimal preventive effects. In one trial, already 10–18 sessions of CCT showed beneficial effects on processing speed and cognitive impairment [37]. As the main findings from FINGER indicate that the multi-domain intervention, including CCT, is effective in improving cognitive function and reducing cognitive decline [18], it may be that even smaller amounts of CCT are sufficient when combined with other interventions.

The strengths of this study include the large sample size and comprehensive data collection on possible predictors. Participants were drawn from the general population, and they are representative of a large portion of this age group [38]. Most previous studies are either small scale and/or conducted among selected populations or volunteers. Unlike most related studies that focus on individuals already having major cognitive problems, this study included only high-risk individuals. The participants did not have substantial cognitive problems at study entry, and, overall, the cognitive test performance was improved during the 2-year trial, but with some variability across participants [18]. Another strength of this study is that adherence was not based on self-reports, but on computer-use logs.

Some limitations should be noted. First, although we examined a large number of possible predictor variables, there are other factors that may be important to consider, for example, self-efficacy or personality traits [11,39]. Second, although our participants are representative of a large part of an older population, due to the inclusion criteria, persons with either high or very low cognitive performance were not included [38]. Third, we could identify predictors of adherence, but we could not investigate the exact reasons why an individual chose not to adhere. Fourth, complete data was missing for 11% of the participants. The amount of missing data was quite limited. The persons with missing data had completed less CCT but were otherwise similar to the participants with full data in most of their characteristics. If anything, the missing data might lead to diluted association between CCT adherence and study expectations, sex, and processing speed.

Our study indicates a large variation in adherence to a long-lasting CCT among older adults with an increased risk of dementia. Important determinants of adherence were identified. Facilitating computer use (both access and skills), providing extra support for those with memory problems or hesitating with their participation may help in conducting future population-based CCT studies and implementing CCTs in practice.
